# Supplementary material for: Distinct Tumor-Associated Macrophage Signatures Shape the Immune Microenvironment and Patient Prognosis in Renal Cell Carcinoma
Source: Cells. 2025 Nov 6;14(21):1740. doi: 10.3390/cells14211740 (PMC12610877; doi:10.3390/cells14211740)
Supplement: Supplementary file 1 [file cells-14-01740-s001.zip › Supplemental Information/Supplemental document.docx]

**Supplemental Figure 1. The Independent method, BASE, validates the TAM signatures with important immune cells.** (A) Correlation Between TAM Signatures and KIRC Immune Cells: This heatmap demonstrates the correlation between the 8 TAM signatures and six major immune cell types from the KIRC dataset. Except TAM5 and 6, positive associations are observed, particularly with macrophages, NK cells, and CD8+ T cells, suggesting TAM involvement in RCC immune regulation. (B) PC1 Correlation with Immune Cells: Correlation analysis validated the association between PC1 score and immune cell populations derived from independent deconvolution methods (KIRC immune cells). PC1 showed strong correlations with macrophages, CD8+ T cells, and NK cells, supporting the interplay between the selected TAMs and these key immune effector cells. (C) Survival plot of the TAM1,2,3,4,7,8 signatures. (D) Survival plot of the PC1.
